# Pathological studies on postvaccinal reactions of Rift Valley fever in goats

**DOI:** 10.1186/1743-422X-6-94

**Published:** 2009-07-06

**Authors:** Samia Ahmed Kamal

**Affiliations:** 1 Animal Health Research Institute, Dokki, Giza, Egypt

## Abstract

RVF live attenuated vaccine (Smithburn strain) was evaluated by using goats as experimental animal. The results indicate that this vaccine cause severe deleterious pathological changes in liver especially in kids and causing abortion in pregnant does. The virus was seen to be propagated inside hepatic cells forming intranuclear inclusions which was also seen by E.M. Viral antigens were detected in hepatic cells, gall bladder, endothelial lining of blood vessels, leukocytes, kidneys and heart by using immunoflourescent technique. It could be concluded that the use of live attenuated vaccine of RVF (Smithburn strain) for immunization of live stock is not safe in Egypt as it considered an endemic area.

## Introduction

Rift Valley Fever (RVF) is a febrile disease that affected livestock and humans, transmitted by mosquitoes and caused by a virus (genus: Phlebovirus, family: Bunyaviridae) that can persist in nature [1]. The virus was first isolated in Kenya in 1930 [2]. Until 1977, Rift Valley Fever disease was geographically limited to Africa, and then it was recorded for the first time in Egypt as an epizootic [3]. The Rift Valley Fever virus is a single stranded RNA with three segments (S, M, and L), each segment is enclosed in a separate nucleocapsid within the virion [4].

Rift Valley Fever (RVF) virus causes abortion in pregnant animals and a high mortality in young ones and humans especially those suffering other diseases [5]. The most prominent and pathognomonic lesions of RVF virus infection were found in the liver [6]. RVF virus is a hepatotropic virus in vivo and can cause liver necrosis and death in animals [7]. The intranuclear inclusion bodies inside the hepatic cells and the necrotic foci are of diagnostic importance which characterizes RVF infection [8]. The Aim of this Work was to: Evaluate the live attenuated RVF vaccine which was produced locally in Egypt from pathological point of view with discussing the picture of the disease that might result from this vaccine by using young kids, adult does and pregnant does.

## Materials and methods

### Materials

#### 1-Experimental animals

The number of animals used in this study was fourteen (14). They were classified into three groups. The first group was five kids (1.5 month old), the second group was five non pregnant adult does (1 year old) and the third group was four pregnant does (1.5 year old and of three months pregnancy). Meanwhile, the animals were tested serologically against RVF virus to prove that they were completely free from infection and did not contain neutralizing antibodies against RVF virus. Four kids, four non pregnant does and three pregnant does were vaccinated by RVF attenuated vaccine subcutaneously 1 ml/animal.

#### 2-Samples

1-Whole blood. 2-Serum samples. 3-Frozen tissues specimens. 4-Formalin fixed tissues specimens. 5-Glutaraldehyde fixed tissues specimens. 6-Frozen tissues specimens for A.G.P.T.

### Methods

#### 1-Haematological studies

Erythrocytic count (RBCs) was performed using improved neubauer haemocytometer and hayme's solution. The packed cell volume (PCV) was done using the microhaematocrit centrifuge. Total leucocytic count (TLC) was performed using improved neubauer hemocytometer and turkey's solution. Blood films were prepared and stained using Giemsa stain for differential leucocytic count, which was done by Battelement method. All hematological studies were done according to [9]. While haemoglobin (Hb) was determined using test kits of Diamond according to [10]. Whole Blood Clotting Time was done after [11]; protocols require standardization of blood volume, clean glass tubes of a standard size and a water bath (25–37°C). Using whole blood immediately after collection; contact activation of coagulation is initiated by the glass tube.

#### 2-Biochemical Analysis

Aspartate Aminoransferase (AST), Alanine Aminotransferase (ALT) and serum urea were determined according to [12]. Alkaline phosphatase (ALP) was measured according to [13].

#### 3-Viral antigens

The detection of viral antigen was done by indirect immunofluroscent technique on frozen sections and paraffin sections using hyperimmunserum of RVF which prepared in rabbit and anti-rabbit IgG conjugate with fluorescein isothiocyanate according to[14], and also by agar gel precipitating test (AGPT) on organ homogenates according to [15].

#### 4-Histopathological Examination

Formalin fixed specimens were prepared and examined microscopically [16]. Another paraffin sections were stained by PAS procedure (Periodic acid-Schiff) and Phloxine-Tartarzine stain according to [14].

#### 5-Transmission electron microscopy examination

The glutaraldehyde fixed tissues specimens were prepared and examined microscopically by the electron microscope [17].

## Results

### 1-Haematological results

The RBCs count, Hb concentration and PCV% showed a significant decrease than control in all vaccinated animals beginning from second day postvaccination in group 1&2 and started at 3^rd ^day P.V. in group three. Then showed a significant decrease at 3^rd^, 4^th^, 5^th^, 6^th ^and 7^th ^days P.V. in all vaccinated goats than control. The type of anaemia was detected after determination of MCV and MCHC. The results indicated that the type of anaemia was normocytic normochromic at the 2^nd^, 3^rd ^and 4^th ^days P.V. in groups (1&2) and at 3^rd ^and 4^th ^days P.V. in group (3). Then the anaemia type became macrocytic hypochromic at 5^th^, 6^th ^and 7^th ^days P.V. in all groups.

Total leucocytic count (TLC) and differential leucocytic count; there was a significant increase in TLC in 1^st^, 2^nd ^and 3^rd ^day P.V. in group (1) and group (2) and up to 4^th ^day P.V. in group (3) than control. Significant decrease in TLC was recorded at 6^th ^and 7^th ^days P.V. in group (2). Significant decrease in TLC was recorded at 2^nd^, 3^rd ^and 4^th ^weeks P.V. in vaccinated animals of all groups. There was a significant increase in segmented neutrophil at 1^st^, 2^nd ^and 3^rd ^days P.V. in group (1) and group (2) followed by a significant decrease at 6^th^,7^th ^days P.V. and at and 2^nd^,3^rd ^and 4^th ^weeks P.V. in both groups. In group (3) a significant increase in segmented neutrophil was recorded at 1^st^, 2^nd^, 3rd and 4^th ^days P.V. then a significant decrease in segmented neutrophil was recorded at 7^th ^day P.V. and at 2^nd^, 3^rd ^and 4^th ^weeks P.V. There was a significant increase in lymphocyte in group (1) at 1^st ^day P.V. and at 1^st ^and 2^nd ^day P.V. in group (2) and at 1^st^, 2nd and 6^th ^day P.V. in group (3) followed by significant decrease at 2^nd^, 3rd and 4^th ^weeks P.V. in groups (1) and (2) but at 3^rd ^and 4^th ^weeks P.V. in group (3).

### 2-The biochemical results

#### a-Whole blood clotting time(WBCT)

There was a significant prolongation of the whole blood clotting time than control in all vaccinated animals with differences according to the age and physiological states (pregnant or not). The highest level of clotting time was observed in the pregnant does (7.2 minutes) at 7^th ^day P.V. Then gradually decreased in 2^nd^, 3rd and 4^th ^weeks P.V.

#### b-Clinicopathological results

##### Group (1)

a- AST and ALT in young kids showed an increase beginning from 2^nd ^day postvaccination (P.V.) and reach its peak at 7^th ^day P.V. & 6^th ^day P.V. respectively and was still high at 2^nd^, 3^rd ^& 4^th ^weeks P.V. than control. b- ALP in young kids showed an increase beginning from 1^st ^day P.V. and reaches its peak at 6^th ^day P.V. and still was high at 2^nd^, 3^rd ^and 4^th ^weeks P.V. than control.

##### Group (2)

a- AST and ALT in adult doe showed an increase beginning from 2^nd ^day postvaccination respectively and reach its peak at 5^th ^day and 6^th ^day postvaccination respectively and was still high at 2^nd^, 3^rd ^and 4^th ^weeks P.V., b- ALP in adult doe showed increase beginning from 1^st ^day P.V. and reaches its peak at 5^th ^day P.V. and was still high at 2^nd^, 3^rd ^and 4^th ^weeks P.V.

##### Group (3)

a- AST and ALT in pregnant does showed increase beginning from 2^nd ^day postvaccination and reach its peak at 6^th ^day and 5^th ^day P.V. respectively and was still high at 2^nd^, 3^rd ^and 4^th ^weeks P.V., b- ALP in pregnant does showed increase beginning from 1^st ^day P.V. and reaches its peak at 6^th ^day P.V. and was still high at 2^nd^, 3^rd ^and 4^th ^weeks P.V.

### 4- Histopatholoical Results

#### Group (1) Kids

**Figure 1:**
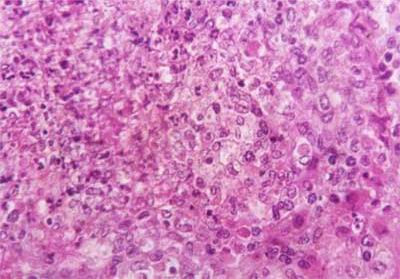
**Liver of kid in group-1, animal sacrificed one week P.V., showing necrotic focus with Councilman-like bodies inside necrotic and degenerated hepatocytes**. (H&E × 400).

**Figure 2:**
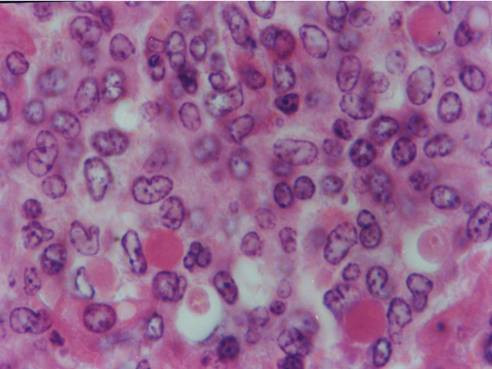
**Liver of kid in group-1, animal sacrificed one week P.V., showing Councilman-like bodies appeared as oesinophilic masses surrounded by hallo zone (green arrow) and Russell's bodies**. (H&E × 1000).

**Figure 3:**
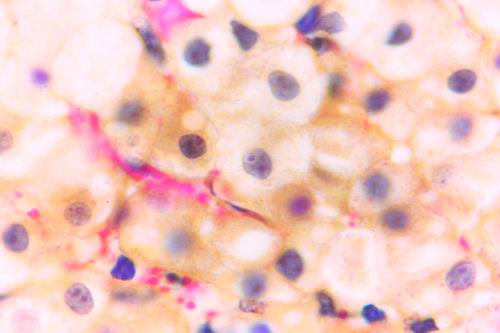
**Liver of kid in group-1, animal sacrificed one week P.V., showing the intranuclear inclusion bodies appeared rounded and surrounded with hallo inside degenerated hepatocytes nuclei**. (Phloxine-Tartrazine stain × 1000).

**Figure 4:**
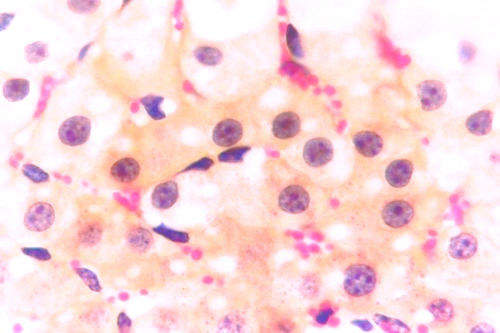
**Liver of kid in group-1, animal sacrificed one week P.V., showing the intranuclear inclusion bodies appeared as one, two or three inclusions**. (Phloxine-Tartrazine stain × 1000).

**Figure 5:**
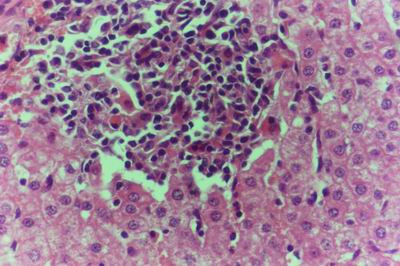
**Liver of kid in group-1, animal sacrificed one week P.V., showing apoptosis affecting the individual cells**. (H&E × 400).

**Figure 6:**
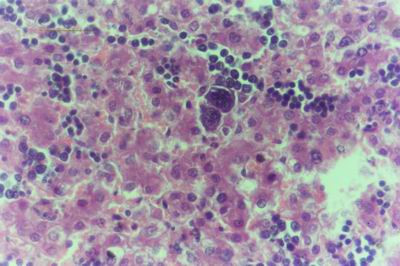
**Liver of kid in group-1, animal sacrificed one week P.V., showing megakaryocytes in the liver parenchyma and their nuclei were hyperchromatic**. (H&E × 400).

**Figure 7:**
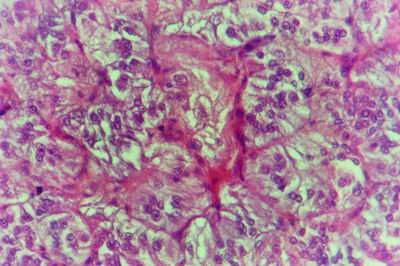
**The adrenal gland of kid in group-1, animal sacrificed one week P.V., showing hyperplastic proliferation in zona fasiculata**. (H&E × 400).

**Figure 8:**
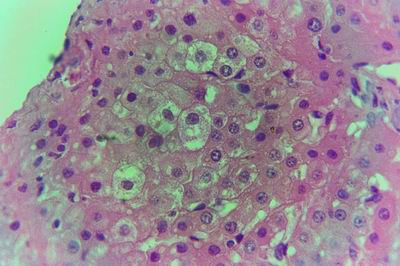
**Liver of kid in group-1, animal sacrificed two weeks P.V., showing diffuse vacuolar degeneration of hepatocytes**. (H&E × 400).

**Figure 9:**
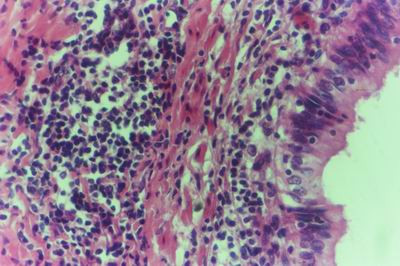
**Liver of kid in group-1, animal sacrificed two weeks P.V., showing hyperplasia of the epithelial lining of the bile duct with severe lymphocytic infiltration**. (H&E × 400).

**Figure 10:**
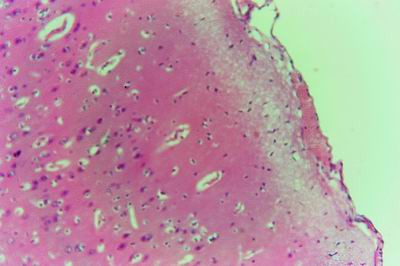
**Brain of kid in group-1, animal sacrificed two weeks P.V., showing blood vessels congested with perivascular oedema**. (H&E × 100).

**Figure 11:**
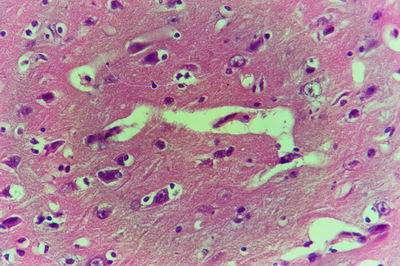
**Brain of kid in group-1, animal sacrificed two weeks P.V., showing blood vessels congested with perivascular oedema**. (H&E × 400).

**Figure 12:**
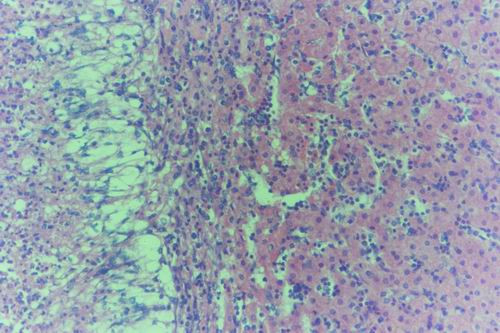
**Liver of kid in group-1, animal sacrificed three weeks P.V., showing large number of mononuclear cells infiltration together with necrosis and loss of cellular details (washed out appearance)**. (H&E × 200).

**Figure 13:**
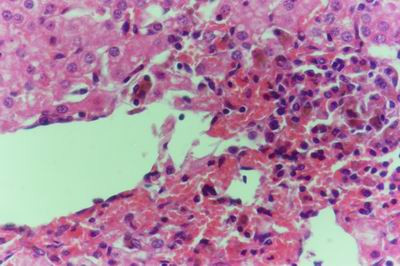
**Liver of kid in group-1, animal sacrificed four weeks P.V., showing centrolobular hepatic necrosis and haemorrhage around the central vein with brownish haemosiderin pigment deposition**. (H&E × 400).

Kid sacrificed one week postvaccination showed that, the liver has the most prominent lesions (necrogranulomes). These granulomes were focal areas of necrosis invaded by macrophages, lymphocytes and plasma cells. These necrotic foci were scattered allover the entire hepatic lobules. The necrotic foci have necrotic debris in the centre and the border showed signs of necrosis and degenerated hepatic cells. Councilman's-like bodies were seen inside the cytoplasm of swollen, degenerated and necrotic hepatocytes. These bodies appeared spherical, refractile and eosinophilic hyaline masses (Fig. 1). In some cases, the necrotic foci contained extracellular and intracellular spherical eosinophilic refractile bodies seen among the inflammatory cells (Russell's bodies) (Fig. 2). The hepatic parenchyma also showed degeneration and necrosis in other parts (paracentral necrosis). Nearly all the subcapsular hepatocytes appeared swollen degenerated and contained intranuclear inclusion bodies. The inclusion bodies were confirmed by positive reaction to Phloxine-Tartrazine stain. The detected inclusions sometimes appeared rounded and surrounded with hallo zone in degenerated nucleus (Fig. 3). The detected inclusion appeared as one, two or three inclusions inside the nuclei (Fig. 4). Some areas in the hepatic lobules showed disorganization of the hepatocytes in which the cells were not arranged in cords. The hepatic cell plates have been destroyed (lobular disarray) and the surviving hepatocytes were forming rounded hyperplastic nodules without lobular arrangement. Apoptosis was observed near the necrotic foci and nearly allover the entire hepatic lobules, affecting the individual cells (Fig. 5). The endothelial lining of the hepatic sinusoids showing signs of necrosis and degeneration & sinusoidal dilatation were observed. The bile ducts showed hyperplastic proliferation of their epithelial lining. In the liver megakaryocytes were seen inside hepatic sinusoids and their nuclei were hyperchromatic accompanied by sinusoidal leuckocytosis (Fig. 6). The lymph nodes showed hyperplastic activation of lymphocytes in the form of follicular and paracortical hyperplasia that manifested by numerous large lymphoblasts in the paracortical zone with evidence of mitosis and presence of intranuclear inclusion bodies. The spleen also exhibited lymphocytic activation of the white pulp. The kidneys showed areas of necrosis. The proximal convoluted and distal tubules were suffering from degenerative and necrotic changes. Intranuclear inclusion bodies were demonstrated inside the tubular epithelium as demonstrated by phloxine-Tartrazine stain. The adrenal gland showed hyperplastic activation in zona fasiculata (Fig. 7) and some necrotic changes in the medulla were also seen. Kid sacrificed two weeks postvaccination showed diffuse vacuolar degeneration of hepatocytes (Fig. 8) with kupffer cells activation. Minute necrotic foci were seen in the hepatic parenchyma invaded by macrophage and lymphocytes. The endothelial lining of the hepatic sinusoids showing signs of necrosis and degeneration & sinusoidal dilatation were observed. Councilman's-like bodies were seen in the degenerated hepatocytes. Intranuclear inclusion bodies were detected inside degenerated hepatocytes adjacent to areas of coagulative necrosis. Hyperplasia of the epithelial lining of the bile ducts and lymphocytic infiltration were seen in the portal areas (Fig. 9). The lymph nodes and spleen revealed lymphocytic depletion and the kidneys exhibited necrotizing changes in the proximal and distal convoluted tubules and the adrenal glands showed hyperplasia of zona fasciculata and degenerative changes in the medulla. The brain blood vessels were congested and engorged with blood with perivascular oedema (Fig. 10 &11). Neuronal degeneration and necrosis was seen accompanied by astrocytic oedema, microglial proliferation and neuronophagia. Kid sacrificed three weeks postvaccination showed large necrotic foci with destructed center surrounded by macrophages, lymphocytes and necrotic hepatocytes. Intracytoplasmic inclusion-like bodies surrounded by hallo zone (Councilman's-like bodies) were seen in the degenerated hepatocytes. Near these necrotic foci, abnormal cellular growth accompanied by dilated blood vessels and haemorrhages were observed. The endothelial lining of the hepatic sinusoids showing signs of necrosis and degeneration & sinusoidal dilatation were observed. Severe hemorrhages were seen in some areas of the hepatic lobules that suffer from degeneration and necrosis. Sinusoidal dilatation and disconfiguration of the hepatic parenchyma was seen. Some bile ducts showed hyperplasia of the epithelial lining. Large number of mononuclear cells infiltration was observed in some parts of hepatic tissue together with necrosis and loss of cellular details that gave hepatocytes washed out appearance (Fig. 12). Kid sacrificed four weeks postvaccination showed severe centrolobular hepatic necrosis (periacinar necrosis) and severe haemorrhages around the central veins with hemosiderin pigments deposition (Fig. 13). The subcapsular hepatic parenchyma showed swollen hepatocytes with coagulative necrosis. The degenerated and swollen hepatocytes contained numerous intracytoplasmic inclusion-like bodies that appeared as eosinophilic rounded and well-circumscribed masses of different sizes surrounded by hallo zone (Councilman's-like bodies). The kupffer cells were highly proliferated. Some hepatocytes undergo apoptosis and appeared shrinked, differed in shape from the adjacent hepatocytes with condensed cytoplasm, detached from other adjacent hepatocytes. Nuclear chromatin was condensed, clumped and the apoptotic bodies were seen. The endothelial lining of the hepatic sinusoids showing signs of necrosis and degeneration & sinusoidal dilatation were observed. Some bile ducts undergo massive destruction of epithelial lining and invaded by large numbers of lymphocytes and macrophages. The myocardium showed areas of hemorrhages around the dilated and necrotic blood vessels. Zenker's necrosis and focal infiltration with macrophages and lymphocytes were observed. The spleen showed severe lymphocytic depletion. The kidneys showed signs of necrosis in the renal corpuscles and the renal tubular epithelium showed intranuclear inclusion bodies surrounded by hallo zone. The adrenal glands showed hyperplastic changes in zona fasciculata and medulla. The brain blood vessels were slightly congested.

#### Group (2) Adults non pregnant

**Figure 14:**
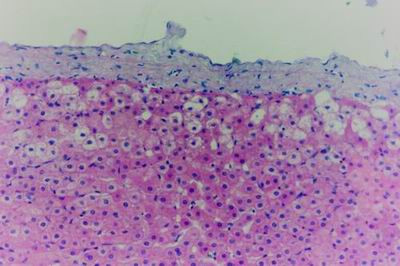
**Liver of adult goat in group-2, animal sacrificed one week P.V., showing subcapsular hepatic degeneration**. (H&E × 200).

**Figure 15:**
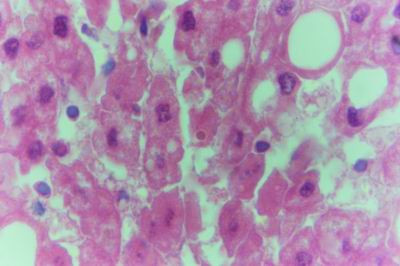
**Liver of adult goat in group-2, animal sacrificed one week P.V., showing swollen hepatocytes contained intracytoplasmic inclusion-like bodies surrounded by a hallo (Councilman-like bodies)**. (H&E × 1000).

**Figure 16:**
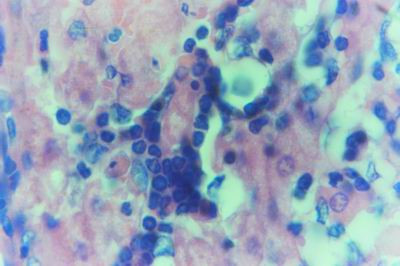
**Liver of adult goat in group-2, animal sacrificed two weeks P.V., showing hepatic necrosis and disconfiguration of the hepatic parenchyma together with macrophages aggregation around necrotic hepatocytes**. (H&E × 1000).

**Figure 17:**
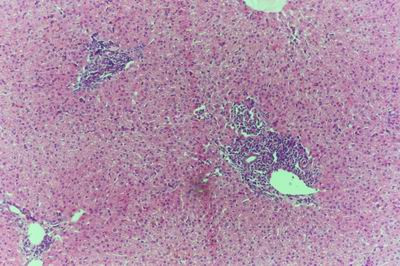
**Liver of adult goat in group-2, animal sacrificed four weeks P.V., showing lymphocytic aggregations around the portal areas**. (H&E × 100).

**Figure 18:**
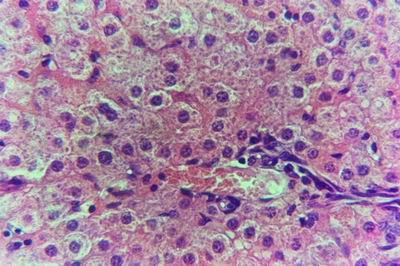
**Liver of adult goat in group-2, animal sacrificed four weeks P.V., showing hepatocytes with vacuolation and necrosis**. (H&E × 400).

**Figure 19:**
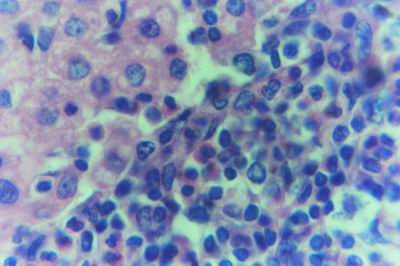
**Liver of adult goat in group-2, animal sacrificed four weeks P.V., showing necrotic area invaded with macrophages and lymphocytes**. (H&E × 1000).

Doe sacrificed one week postvaccination showed diffuse centrolobular coagulative necrosis of liver parenchyma (periacinar). Sinusoidal dilatation that causes disconfiguration of the liver parenchyma and thrombus formation in the hepatic artery were also demonstrated. The hepatic cells under the liver capsule were swollen and undergo degeneration and some of them undergo necrotic changes (Fig. 14). Necrotic foci were found in some hepatic lobules near the central veins (midzonal). The degenerated hepatocytes that found adjacent to the necrotic areas contained eosinophilic intranuclear inclusion bodies and their cytoplasm contained fine granules which stained positive with Phloxine-Tartrazine stain. The swollen and degenerated hepatocytes contained intracytoplasmic bodies surrounded by a hallo zone (Councilman's-like bodies) (Fig. 15). Haemorrhages were detected in areas around the central veins. Some bile ducts were infiltrated with lymphocytes and others were hyperplastic and its epithelium was elongated and branched inside the lumen. The endothelial lining of the hepatic sinusoids showing signs of necrosis and degeneration & sinusoidal dilatation were observed. The lymph nodes appeared to be hyperplastic and the lymphoid follicles revealed activation that manifested by large lymphoblast suffering from mitosis especially in paracortical area (paracortical hyperplasia). The spleen also revealed hyperplastic white pulp. The kidneys showed nephrosis of the renal tubules. The lining epithelium of proximal tubules showed pyknotic and lysed nuclei beside the degenerative and necrotic changes that observed in the adjacent tissue. Intranuclear oesinophilic inclusion bodies were demonstrated inside the tubular epithelium as demonstrated by Phloxine-Tartrazine stain. The adrenal glands were hyperplastic particularly in zona fasciculata with some necrotic changes in the medulla. Doe sacrificed two weeks postvaccination showed that the hepatic lesions were centrolobular coagulative necrosis (periacinar), preceded by granularity of the hepatocytes cytoplasm. Areas of haemorrhages around the central veins were noticed. Kupffer cells were seen engulfing hemosiderin pigments. Moderate number of apoptotic cells were seen allover the hepatic lobule. The endothelial lining of the hepatic sinusoids showing signs of necrosis and degeneration & sinusoidal dilatation were observed. Disconfiguration of the hepatic parenchyma was seen in some areas together with macrophage aggregations around necrotic hepatocytes (Fig. 16). Pyknotic nuclei of hepatic cells with karyorrhexis and karyolysis were also seen in the necrotic areas around the central veins. The hepatocytes that lying under the hepatic capsule were swollen and contained eosinophilic intracytoplasmic bodies of different sizes and was surrounded by hallo zone (Councilman's-like bodies). Some bile ducts showed necrotic epithelium and others showed hyperplastic overgrowth with vesicular elongated epithelium and lymphocytic infiltration. The myocardium showed areas of necrosis (Zenker's necrosis) accompanied by haemorrhages and lymphocytic infiltration. Depletion of lymphocytes from the spleen and lymph nodes associated with subcapsular macrophages in lymph nodes. The kidneys showed necrosis in the renal corpuscles and tubules. The adrenal glands showed hyperplasia in the cortex and necrosis in the medulla. The brain showed perivascular and astrocytic edema, focal gliosis and lymphocytic infiltration in the Virchow-Robin spaces (cuffing), microglial proliferation and some neurons were necrotic and invaded by microglia (neuronophagia). Doe sacrificed three weeks postvaccination showed centrolobular hepatic necrosis (periacinar necrosis). The haemorrhages were seen near the necrotic areas with discontinued blood vessels (central veins). Apoptotic cells were seen in the hepatic parenchyma around and inside the necrotic areas. The bile ducts showed macrophages and lymphocytes around it. Some bile ducts showed severe hyperplastic proliferation. The myocardium, spleen, lymph nodes, kidneys, adrenal glands and brain showed similar but more severe necrotic changes than that mentioned before at two weeks postvaccination. Doe sacrificed four weeks postvaccination showed more severe destruction of the hepatic parenchyma and necrosis accompanied by lymphocytic infiltrations (Fig. 17). Almost all the hepatic cells were swollen and contained intracytoplasmic bodies (councilman's-like bodies). Some areas in the hepatic lobules were undergoing vacoulation and necrosis (Fig. 18). The necrotic areas at this stage appeared with large number of macrophages and lymphocytes (Fig. 19). The bile ducts were massively destructed and some of them showed severe hyperplasia.

**Figure 20:**
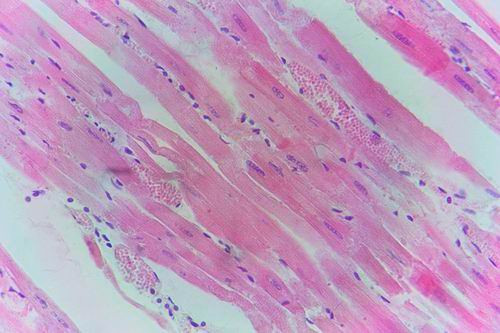
**Myocardium of adult goat in group-2, animal sacrificed four weeks P.V., showing congestion of myocardial blood vessels with few lymphocytic infiltrations**. (H&E × 200).

The myocardium showed Zenker's necrosis with severe lymphocytic infiltration and its blood vessels endothelium showed necrosis and discontinuation accompanied by areas of haemorrhages (Fig. 20). The spleen, lymph nodes, kidneys, adrenal glands and brain showed more exaggerated changes similar to those observed at three weeks postvaccination.

#### Group (3) Pregnant Does

**Figure 21:**
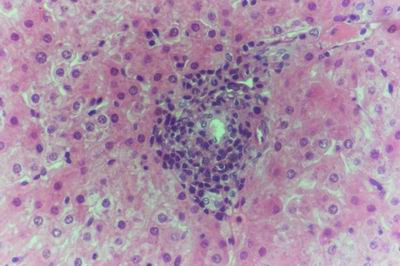
**Liver of aborted doe in group-3, animal sacrificed 10 days P.V., showing periportal focal necrosis with invasion of portal area with lymphocytes**. (H&E × 400).

**Figure 22:**
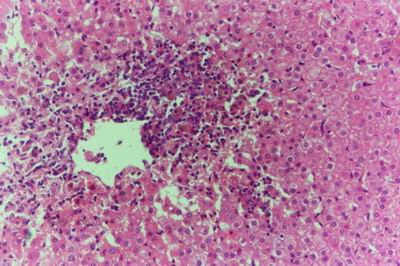
**Liver of aborted doe in group-3, animal sacrificed 10 days P.V., showing periacinar necrosis and haemorrhages with haemosiderin deposition inside infiltrated macrophages**. (H&E × 200).

**Figure 23:**
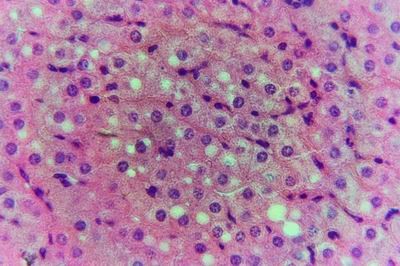
**Liver of aborted doe in group-3, animal sacrificed 28 days P.V., showing Kupffer cells proliferation and vacuolar degeneration**. (H&E × 400).

**Figure 24:**
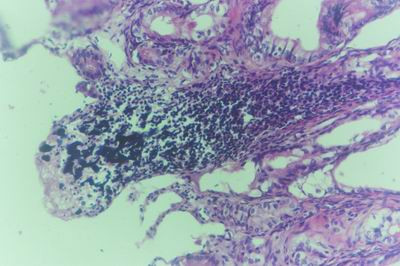
**Uterus of aborted doe in group-3, animal sacrificed 10 days P.V., showing the necrotic endometrial lining with areas of necrosis and lymphocytic infiltration in the tunicae**. (H&E × 400).

**Figure 25:**
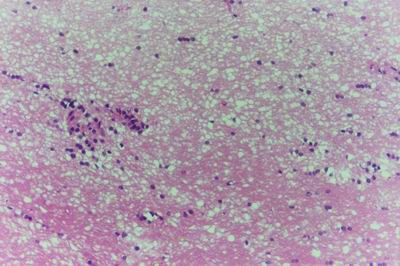
**Brain of aborted foetus in group-3, 28 days P.V., showing gliosis**. (H&E × 200).

The hepatic lesions in this group were quite similar and characterized by periacinar necrosis (Fig. 21). The hepatocytes around central veins were completely necrotized and the peripheral cells contained Councilman's like bodies inside cytoplasm also intranuclear inclusion could be seen. Extravasated RBCs aggregated around central veins and portal areas were heavly infiltrated with macrophages and lymphocytes (Fig. 22). Thrombus was found inside another cenral vein and was infiltrated by lymphocytes. Kupffer cells proliferation and vacular degeneration were also seen (Fig. 23). The lymph nodes and spleen were depleted from mature lymphocytes with necrosis. The uteri showed necrotic endometrial lining with areas of necrosis in the tunica muscularis and lymphocytic infiltration (Fig. 24). The endometrial blood vessels showed necrotic endothelial lining accompanied by areas of haemorrhages. The renal tubules showed degeneration and necrosis. The adrenal glands showed necrotic cells in the medulla and hyperplasia in zona fasciculata. The brain showed more severe necrotic changes in the neurons with microgliosis and lymphocytic infiltration in the Virchow-Robin spaces. Astrocytic and perivascular oedema were also seen. The aborted and born foeti were showed severe hepatic necrosis (pan-necrosis) accompanied by lymphocytic infiltration. The swollen and degenerated hepatocytes contained intracytoplasmic bodies surrounded by a hallo zone (Councilman's-like bodies) and also some hepatocytes contained inclusion bodies confirmed by Phloxine-Tartrazine stain. The renal tubules were degenerated and some times appeared necrosed. The brain showed meningoencephalitis, oedema and gliosis (Fig. 25).

### 4-Results of electron microscopic studies

**Figure 26:**
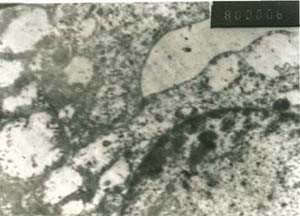
**Liver of adult goat in group-2, animal sacrificed one week P.V., showing hepatocyte with swollen and destructed mitochondria and viral particles inside their cytoplasm**. (E.M. × 8000).

The hepatic cells in young vaccinated kids (group no.1) showed indentation of the nuclear membrane and margination and disintegration of the chromatin. The hepatic cells of vaccinated adult goats (group no.2) showed swollen mitochondria and destructed cytoplasm, (Fig. 26). Some hepatic cells of vaccinated pregnant does (group no.3) revealed condensed chromatin on the nuclear membranes and others revealed concentrated chromatin inside the nucleus accompanied by destructed cytoplasmic organelles. In vaccinated adult goat (group no.2) the proximal convoluted tubules showed necrotic and destructed nucleus with lysed nuclear membrane, fragmented chromatin and lysed cytoplasmic organelles. The microvilli were short and necrotic.

### 4-Immunofluorescent microscopic results

**Figure 27:**
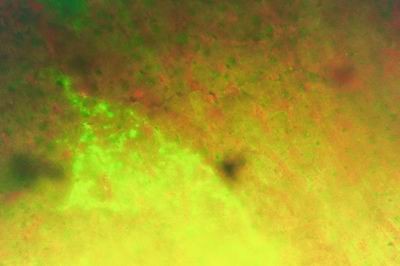
**Liver of kid in group-1, animal sacrificed one week P.V., showing hepatocytes with strong positive fluorescent stain**. (IFA & Evan's blue × 400).

**Figure 28:**
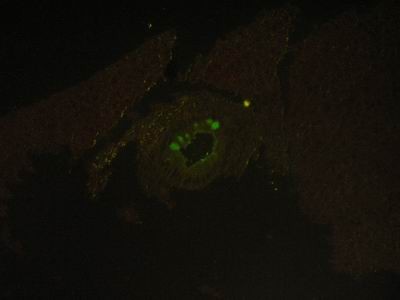
**Liver of kid in group-1, animal sacrificed one week P.V., showing bile duct with strong positive fluorescent stain in the bile duct epithelium (intracytoplasmic)**. (IFA & Evan's blue × 400).

**Figure 29:**
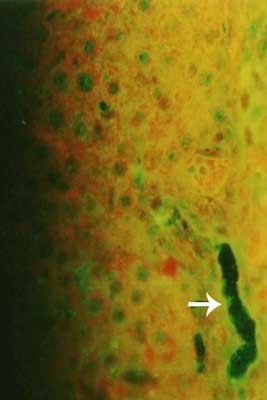
**Liver of adult goat in group-2, animal sacrificed two weeks P.V., showing hepatocytes and endothelium of blood vessels with strong positive fluorescing reaction**. (IFA × 400).

**Figure 30:**
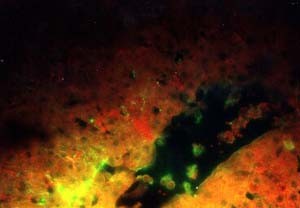
**Liver of kid in group-1, animal sacrificed one week P.V., showing central vein with strong positive fluorescing reaction inside the cytoplasm of the white blood cells**. (IFA & Evan's blue × 400).

**Figure 31:**
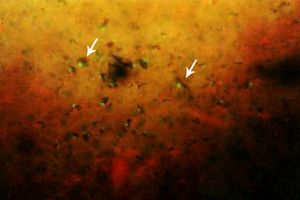
**Liver of adult goat in group-2, animal sacrificed two weeks P.V., showing strong positive fluorescing reaction in the kupffer cells**. (IFA × 400).

The liver in control group gave negative results by showing only the Evan's blue stain reaction. The liver in all vaccinated groups gave positive reaction (Fig. 27). The bile ducts gave strong and characteristic reaction as the antigen appears inside the cytoplasm of its epithelium in all bile ducts (Fig. 28) and also the viral antigen was also detected in the endothelium of the blood vessels (Fig. 29). Strong positive fluorescing reactions were detected inside white blood cells in central vein and in the areas of haemorrhages around this vein (Fig. 30). The proliferated kupffer cells also gave strong and characteristic fluorescing reaction, (Fig. 31). The myocardium in control group gave negative results by showing only the Evan's blue stain reaction. Strong positive fluorescing reactions were detected in the myocardium.

### 5-Agar gel precipitation test (AGPT)

The detection of RVF virus antigen in organs of vaccinated goats with the live attenuated RVF vaccine by agar gel precipitation test (AGPT) on organ homogenates (liver, spleen, kidneys, brain, myocardium and lymph nodes) had given positive reaction in groups 1 and 2 at 1^st^, 2nd and 3^rd ^days and 4^th ^weeks P.V. and in group 3 (pregnant does) at abortion and parturition either from does or dead or stillbirth foeti.

### 6-ELISA

#### Group-(1)

**Kids**: Gave positive results from fourth day postvaccination and reached its highest level at 15^th ^day P.V. (0.782) and persist at a high level till the end of the experiment (4 weeks P.V.).

#### Group-(2)

**Adult does**: Gave positive results from third day postvaccination and reached its highest level at 20^th ^day P.V. (1.006) and persist at a high level till the end of the experiment (4 weeks P.V.).

#### Group-(3)

**Adult pregnant does**: Gave positive results from 3^rd ^day P.V. and and reached its highest level at 20^th ^day P.V. (0.820) and persist at a high level till the end of the experiment (55 days P.V.).

## Discussion

In the present work we tested the live attenuated RVF vaccine (Smithburn strain) in kids, adult and pregnant goats to investigate the adverse effects induced by the vaccine.

Abortions and parturition of dead foeti were additional signs in group three and occurred after sudden and sharp rise in body temperature (41°C). The abortion takes place in two pregnant does out of three. The 1^st ^one was aborted after 10 days postvaccination and the other pregnant one was aborted after 28 days postvaccination. In this concern, [18] mentioned that during the epidemic of Rift Valley Fever that broke out in Egypt in 1977, abortions occurred at any stage of pregnancy after a sharp rise of body temperature accompanied by bloodstained nasal discharge. [19] Mentioned that the inoculation of RVF virus in pregnant ewes resulted in four abortions after 4–12 days with retained placenta.

The results suggested that the causes of abortions and parturition of dead foeti were primarily the direct effect of RVF virus on the genital organs of the pregnant does by causing necrotic changes in the uteri and secondary due to death of the foeti resulted from the infection of the foeti by the RVF virus and propagation of the virus inside the liver of the foeti (RVF antigens in the livers of the aborted and dead foeti were detected by immunoflurescent technique).

The clinical pathological result revealed normocytic normochromic anaemia. [20] Reported that RVF infection in sheep resulted in a significant decrease in RBCs at 1 to 7 days postinfection. [21] Found that the experimental infection of RVF virus in sheep, caused fall of RBCs during the course of the disease.

However, from histopathological point the anaemia was haemorrhagic and this was indicated by widespread haemorrhages in most organs (liver, kidneys, epicardium, endocardium and brain). This haemorrhage may be attributed to three reasons; vascular damage which confirmed by viral antigen in endothelium lining of blood vessels by immunofluorescing staining or; consumption of clotting factors which indicated by massive hepatic necrosis of all vaccinated animals or; thrombocytopenia which may be occurred due to adherence of thrombocytes on damaged endothelium blood vessels and this was proved by extramedullary haematopoiesis in which megakaryocytes observed in hepatic sinusoids, lymph nodes and splenic sinusoids [22].

The clinical results also revealed a significant increase in TLC in the 1^st ^three days in all vaccinated groups and this was resulted from neutrophilia and lymphocytosis and such finding could be attributed to the ability of vaccine to evoke the immune response of animals by stimulation and activation of bone marrow for neutrophilia and lymph nodes for lymphocytosis and this was confirmed histologically by follicular and paracortical hyperplasia of lymph nodes.

On the other hand, decrease in TLC was observed by 2^nd ^week P.V. and this leucopenia was resulted either from neutropenia due to bone marrow affection or from lymphopenia which may be resulted from lymphocytic depletion of lymph nodes and spleen. Our results were concomitant with [23] who mentioned that leucopenia and lymphopenia resulted in case of RVF virus infection while leucocytosis and lymphocytosis resulted from vaccination. Similar findings were observed by [24] and [21].

The results revealed prolongation of the blood clotting time in all vaccinated animals and the highest level of delayed clotting time was observed in the pregnant does (7.2 minutes) at 7^th ^day postvaccination. Our findings were in agreement with [25] who mentioned that one of the main functions that have been deteriorated by RVF virus infection was the coagulation factors of the blood as demonstrated from the significant prolongation of the clotting time. [26] Reported that clotting factors produced by the hepatocytes include fibrinogen, prothrombin and other factors. Consequently, decreased functional hepatic mass may result in a diminishing of clotting factor activity in proportion to the degree of hepatocellular damage, leading to prolonged coagulation times and possible bleeding tendencies. Loss of greater than 70 to 80% of the functional hepatic mass is considered sufficient to cause a clinical coagulopathy.

The results also revealed that there were increases in levels of AST, ALT and ALP in all vaccinated animals. This may resulted from hepatic damage and this was in agreement with [27] who stated that natural infection of RVF virus in sheep caused a significant increase in level of AST and ALT due to liver necrosis caused by RVF virus.

Liver was the most affected organ in all vaccinated groups. The lesions were more obvious and severe in kids than adults. This was in accordance with [28] who stated that the hepatic necrosis in older animals was slightly less extensive than young kids. There were different types of necrosis, periacinar, midzonal, paracentral or even massive necrosis (pan-necrosis) of most hepatocytes. The necrosis could be attributed to either cytotoxic effect of virus or ischemia that resulted from hepatic haemorrhages due to the damage of the endothelial lining of hepatic sinusoids. However, [29] mentioned that the frequent paracentral location of the liver lesions is suggesting the association of the changes with anoxia. This finding was in agreement with [30] who mentioned that in livers of lambs infected with Rift Valley Fever virus naturally, showed scattered grayish-white foci of 1–2 mm in diameter and hemorrhages of varying sizes were seen throughout.

Intranuclear inclusion bodies were detected inside the cells of liver and kidneys of all the vaccinated kids, adults and pregnant does. These inclusions were differed in shape and in some cells the chromatin of the affected nuclei were marginated and disintegrated as shown by the electron microscope examination. The intranuclear inclusions were eosinophilic by Phloxine-Tartrazine stain and surrounded by a hallo and founded inside cells which suffering degeneration and necrosis and located inside or near the necrotic areas in the hepatocytes and the epithelial lining of the renal tubules. Our findings were in agreement with [22]. However, [7] mentioned that viruses of Bunyaviridae family encode their structural proteins but moreover Phlepoviruses also encode a nonstructural protein (NSs) in the viral RNA (vRNA) of their S segment. For RVF virus, the NSs proteins were reported to be phosphorylated and to accumulate in the nuclei of the infected cells. Studies concerning genomic replication indicated that continous proteins synthesis was required for replication of the RVF viral genome and these proteins were of viral origin. For the viruses in the family Bunyaviridae the structural protein, nucleocapsid protein (N), would function to regulate replication and would be tempered by the presence of nonstructural proteins (NSs), where they exist.

Recently, [31] mentioned that a better understanding of the factors that govern RVFV virulence and pathogenicity is required, given the urgent need for antiviral therapies and safe vaccines. NSs with anti-IFN activity accumulated in the nucleus. IFN synthesis is regulated by specific transcription factors, including interferon regulatory factor (IRN-3), NF-kappaB, and AP-1. In the presence of NSs, IRF-3 was still activated and moved to the nucleus. So, these results suggest that NSs, unlike other viral IFN antagonists, does not inhibit IFN-specific transcription factors but blocks IFN gene expression at a subsequent step.

The Councilman's-like bodies were seen inside the cytoplasm of swollen, degenerated and necrotic hepatocytes in all vaccinated animals and in the aborted foeti and dead foeti as well. The bodies appeared as intracytoplasmic eosinophilic masses surrounded by hallo zone.

However, in this concern [32] gave description to the Councilman's bodies as small, hyalinous, round or oval eosinophilic inclusions in the cytoplasm of hepatic cells infected with Yellow fever virus and suggested that these bodies represent necrosis around viral particles. He added that these bodies might also see in other form of toxic or viral hepatitis. [29] Mentioned that the affected liver cells with RVF virus were swollen, eosinophilic, hyaline cytoplasm, and pyknotic or fragmented nuclei, their appearance suggesting the Councilman's bodies of yellow fever.

Recently, [22] stated that Councilman's-like bodies were seen in some viral infections and may be due to condensation of cytoplasmic organelles and squestrated from remaining cytoplasm by membranes that fuse with lysosomes (autolysosomes) and may also be derived from other hepatocytes that suffered from apoptosis (apoptotic bodies) and engulfed by remaining hepatocytes.

In the present work, we observed large numbers of apoptotic cells in liver of vaccinated animals by RVF live attenuated vaccine. Our findings are in concurrence with [33] who mentioned that viral infection can induce PCD in various host target cells. Also we agree with [22] who mentioned that RVF virus increases the apoptotic hepatic cells. These findings are in concurrence with [34] who mentioned that Bunyamwera virus nonstructural protein (NSs) counteracts interferon regulatory factor 3 (IRF-3), so leads to the induction of early cell death (PCD).

Russell's bodies were seen inside the necrotic foci in the form of intracellular and extracellular spherical strongly eosinophilic bodies in some cases of group-1 (kids). However, [35] described the so called Russell's bodies as small, spherical hyaline bodies in cancerous and simple inflammatory growth and in degenerating plasma cells. Then, [36] discussed the various theories of Russell's bodies origin. The possibilities included, origin from the lymphocytes, origin in plasma cells with later degeneration, origin from mitochondria of cells, and even an origin from a red blood cell swallowed up by a plasma cell. These bodies were seen within the tissue cells (intracellular) and outside the cells (extracellular). The size of Russell's bodies ranged from barely visible up to "half again" as large as red blood corpuscles. The largest round forms were easily seen microscopically. Recently, [37] mentioned that Russell bodies (RB) are dilated ER cisternae containing condensed immunoglobulins (Ig). As to their biogenesis, it was shown that the synthesis of a mutated Ig, which is neither secreted nor degraded, is sufficient to induce RB formation in cells of different species and histotype. Many disease-linked cases of intralumenal protein accumulation have been described including thyrocytes of congenital goiter patients and hepatocytes of individuals carrying mutated α1 anti-trypsin alleles (PiZ). The correlation between liver disease and PiZ lumenal depositions indicates that the latter can be harmful for cells, but the exact mechanisms that lead to toxicity are not known.

Electron microscopic examination revealed ultrastructure changes in liver and kidneys and viral particles inside hepatocytes cytoplasm. The hepatic cells of vaccinated goats with live attenuated RVF vaccine showed cell with indentation of the nuclear membrane, margination and disintegration of the chromatin. The mitochondria were swollen. Some hepatocytes reveals condensed chromatin on the borders of the nuclei and others revealed more chromatin inside the nuclei. In kidneys the proximal convoluted tubules epithelial cells showed necrotic and fragmented brush borders with signs of necrosis and lyses in their nuclei and other organelles. The distal convoluted tubules showed necrotic microvilli with necrotic and destructed nuclei and lyses of other cell organelles.

Our findings agree with [38] who mentioned that in liver of newborn lamb infected with RVF virus showed ultrastructural changes. Hepatocytes were primarily affected, while inflammatory and structural changes were secondary. [39] Mentioned that the characteristic lesions of RVF were in the liver. Margination of the chromatin in a beaded form or its accumulation in clumps on the nuclear membranes was also observed. In this study we detected the RVF virus antigen in tissues by using the immunoflourescent technique (IFAT) and by agar gel precipitation test (AGPT). The IFAT gave strong positive reactions in organs of all vaccinated animals especially in the liver. The hepatocytes, especially those adjacent to the necrotic foci and to the bile ducts and adjacent to the blood vessels were the most affected and gave the strong fluorescent stain. It was a surprise to found that antigen of RVF virus was seen inside the cytoplasm of the epithelial cells of the bile ducts, nearly in all the examined specimens. These findings suggesting that the RVF virus affects the bile ducts in strong manner and propagates inside its epithelium. However, [40] mentioned that the RVF viral antigen was studied by immunohistochemistry in the liver and was most prominent in the liver and detected in the cytoplasm of hepatocytes which were sparsely scattered throughout the lobules. [41] Mentioned that RVF virus antigen was detected by immunohistochemical analysis in the liver, where positive staining was localized in coalescing foci of hepatocellular necrosis.

From this point of view, RVF virus (Smithburn's strain) could be dessiminated from the vaccinated animals as a result of its propagation and circulation in the blood of the vaccinated animals and resulted in spreading of the RVF virus in the adjacent environment and infected other susceptible animals and even man. These side effects became more dangerous especially in endemic areas as mentioned by [42] who found that genetic exchange occurred between strains from different lineages of RVF virus and this hypothesis emphasizes the risk of generating uncontrolled chimeric viruses by using the live attenuated vaccines in areas of endemicity.

It could be concluded that the use of live attenuated vaccine of RVF (Smithburn strain) for immunization of live stock in Egypt is danger. As the use of live attenuated vaccine of RVF (Smithburn strain) could result in the spreading of RVF virus instead of its eradication. In the present work, the results indicate that this vaccine cause severe deleterious changes in various organs and propagated inside hepatic cells in a manner similar to the infection by the virulent RVF virus. So, the use of live attenuated vaccine (Smithburn's strain) for control RVF virus is not advisable in Egypt as it considered an endemic area for RVF virus.

## Competing interests

The author declares that they have no competing interests.

## Authors' contributions

SAK conceived of the study, and participated in its design and coordination. The author read and approved the final manuscript.
